# The Development of Teachers' and Their Students' Social and Emotional Learning During the “Learning to Be Project”-Training Course in Five European Countries

**DOI:** 10.3389/fpsyg.2021.705336

**Published:** 2021-08-13

**Authors:** Minna Berg, Markus Talvio, Lauri Hietajärvi, Isabel Benítez, Valeria Cavioni, Elisabetta Conte, Francisco Cuadrado, Marco Ferreira, Matej Košir, Baiba Martinsone, Veronica Ornaghi, Irena Raudiene, Daiva Šukyte, Sanela Talić, Kirsti Lonka

**Affiliations:** ^1^Faculty of Educational Sciences, University of Helsinki, Helsinki, Finland; ^2^Department of Methodology for Behavioural Science, University of Granada, Granada, Spain; ^3^“Riccardo Massa” Department of Human Sciences for Education, University of Milano-Bicocca, Milan, Italy; ^4^Department of Communication and Education, Universidad Loyola Andalucía, Seville, Spain; ^5^Higher Institute of Education and Sciences (ISEC), Lisboa, Portugal; ^6^Institute Utrip, Ljubljana, Slovenia; ^7^Department of Psychology, University of Latvia, Riga, Latvia; ^8^Education Academy, Vytautas Magnus University, Kaunas, Lithuania; ^9^Social and Emotional Learning Institute, Vilnius, Lithuania; ^10^Optentia Research Focus Area, North-West University, Vanderbijlpark, South Africa

**Keywords:** social and emotional learning, teacher training and development, social interaction skills, well-being, assessment, intervention

## Abstract

In recent years, the school curricula in many European countries have introduced social and emotional learning (SEL). This calls for the teachers to have SEL competencies. The present study evaluates teachers' and their students' readiness for SEL during an intervention in five European countries. The participants were teachers (*n* = 402) in five European countries; Italy, Latvia, Lithuania, Slovenia, and Spain. The pre- and post-measuring points for both the intervention and the comparison group were at approximately the same time before and after the intervention. Comparison data consisted of 159 teachers in the same countries. The training for the intervention group lasted 16 h for the teachers and a maximum of 16 h for the principles and headmasters. An additional 9 h of further monitoring took place. There were two student groups participating in the study: the age group of 8–11 years (pre puberty) and the age group of 12–15-years (adolescents). Students, whose teachers had participated in the intervention, formed the intervention group (*n* = 2,552). Those students, whose teachers did not participate in the intervention, formed the comparison group (*n* = 1,730). The questionnaire data were collected at the beginning and at the end of the school year for both age groups. The results indicated that there was a favorable development in the intervention group in some of the measured skills among students, but the effects were different for the two age groups. This study adds to both theoretical and practical development of continuing teacher training about SEL and its possible role in reducing problem behavior among the students.

## Introduction

In recent years educational policies decision makers worldwide have shown growing interest toward students' well-being as a facilitator of improved learning (Cohen, 2006; Durlak et al., 2011; Ashdown and Bernard, 2012; Zeidner et al., 2012; Taylor et al., 2017). For example, in council recommendations teaching and learning skills such as self-regulation and other social and emotional skills as a part of twenty-first century skills is seen as means to improve equality among EU-citizens (Council Recommendation of 22 May 2018 on Key Competences for Lifelong Learning Text With EEA Relevance, 2018).

There is some evidence about successful implementation of the international Social and Emotional Learning (SEL) programs to the national context (Talvio, 2014; Gol-Guven, 2016, 2017; Talvio et al., 2016; Cefai et al., 2018; Matischek-Jauk et al., 2018). However, a meta-study analyses on the effectiveness of SEL education programs points out difficulties in transferring educational practices and materials across different countries and stresses out the importance to examine cultural factors that influence the effectiveness of SEL education (Wiglesworth et al., 2016).

In addition to teachers' willingness and the manner with which teaching the SEL skills is transferred into practice, there may be other factors influencing the quality of the implementation in the school level. According to literature (Domitrovich et al., 2008; Humphrey, 2013) the teachers' willingness to implement the skills learned is much defined by how comfortable the teacher feels about implementing the new skills in action. The school climate including an implementation support system that may consist of peer tutoring, a monitoring system as well as leadership support has been reported to increase the fidelity of both the implementation as well as the sustainability of delivering SEL (Humphrey, 2013). Domitrovich et al. (2008) used a multi-level conceptual framework for describing the levels on which implementation quality may be enhanced. They point out that the school level includes components such as the school climate and culture, the resources available as well as the expertise of the staff. This also includes the teachers' possibilities for acquiring peer support for delivering SEL and sharing experiences of success and possible challenges in doing so. The individual level includes the teachers' attitudes toward SEL as well as their willingness and skills for implementing it in their classrooms. The fruitful environment for successful implementation of SEL also includes leadership support. According to Humphrey, support provided by school leadership is a crucial factor for both the sustainability as well as the adaptation of skills in the classroom surroundings.

Accordingly, as it may be worthwhile to pay attention to the quality of the intervention itself and on the implementation process, it may also be worthwhile to pay attention to the timing of the intervention. Some studies suggest that there are some typical SEL programs that work very well with children, but have a poor track record with middle adolescents (Yeager, 2017). The present study concentrates on two age groups of students. The younger group consisted of 8–11 year-old children in pre-puberty and the older group consisted of 12–15 year-old adolescents. These two groups are in developmentally different stages.

Literature shows that (Nolen-Hoeksema et al., 2014) pre puberty is a developmental stage where friendship and social relationships have a growing meaning and interest. They state that beginning from about age five, children develop a sense of obligation to follow the rules handed to them by their parents and teachers. At this age children enjoy games and play that includes agreeing on rules. They also typically look up at the parents and the teacher even though criticism toward adults gradually starts to emerge (Steinberg, 2010). Around the age of 8–9 the children aim at perceiving and understanding the outline of different rules and social schemas and they grow increasingly skilled at taking other people into consideration. Their moral thinking as well as and conscience develop under the guidance of adults and become internalized as personal guidelines (Kagan et al., 1987; Harris, 1995). Consequently, a teacher's toolkit that aims at assessing SEL may help the teacher to pay attention and to guide the students in developing skills for building healthy and ethical relationships at this developmental phase.

The next developmental phase takes place in adolescence at approximately the age of 12–17. At this phase the academic work becomes increasingly complex and demanding and human relations become less stable (Steinberg, 2010). At the same time, the capacity of their brain to process information about emotions undergoes a dramatic transformation (Blakemore and Mills, 2014). Larson et al. (2014) demonstrated that adolescents experienced wide and quick mood swings in this age, and suggested that these mood swings appear to be a natural part of an adolescent peer-oriented lifestyle rather than resulting from stress, lack of personal control or psychological or social maladjustment. They also state that there are indications that these adolescent mood variabilities interfere with capacity for deep involvement, especially in school. The beginning of puberty, which marks the adolescence, causes changes in brain structure as well as in the hormone activity. All these changes can make even minor social challenges, such as peer rejection, difficult to deal with. Consequently, in this age group the teacher's toolkit for assessing SEL may make the students painfully aware of their shortcomings in SEL; there may be a good will, but the regulation of emotions may be challenging. During adolescence, respecting adults may become unimportant and therefore accepting guidance in making changes in one's behavior may become difficult, and problem behavior may start to emerge (Steinberg, 2010). Competence of SEL may help in promoting positive adjustment and in reducing risk for problem behavior (Domitrovich et al., 2017).

Accordingly, school should promote social and emotional learning. Social and emotional skills are taught and learned both intentionally and imperceptibly from peers and teachers, who are both teaching the skills and acting as role models demonstrating the use of skills in action. Therefore, the teachers own skills in social and emotional learning are crucial when teaching the skills to her students (Ferreira et al., 2020). Our EU Erasmus+ project Learning to Be attempted to develop a toolkit with which it would be possible to examine different SEL assessment methodologies in practice in five different European countries. At the same time one of the aims was to investigate first the possible changes in teachers' perceived importance and competence in SEL as well as the possible trend that would show an interrelation between perceived importance and competence in SEL and then during the next phase look at the possible transfer on the students. The aim was to develop a comprehensive and relevant model that would enable positive changes in education policies across Europe. The project aimed at highlighting the necessity to develop positive social and emotional skills among pupils by offering their teachers practical assessing solutions on how social and emotional learning could be integrated into existing education systems as well as providing policy recommendations for supporting social, emotional learning at schools. We also took into account that the challenges for the students in two age groups were different.

### What Is Social and Emotional Learning

SEL is the process by which each student develops their capacity to integrate thought, emotion and behavior in order to achieve and accomplish important social tasks. In this sense, individuals develop skills that allow them to recognize, express and manage emotions, build healthy relationships, establish positive goals, and respond to personal and social needs (Lemerise and Arsenio, 2000; CASEL, 2005). SEL fosters the use of various cognitive and interpersonal skills to achieve relevant goals, both socially and developmentally (Zins et al., 2007). Research indicates that people with solid social and emotional skills are better able to cope with everyday challenges and benefit academically, professionally, and socially (Domitrovich et al., 2017). According to the CASEL (2005), SEL is composed by five key competences namely self-awareness, self-management, social awareness, relationship skills, and responsible decision-making. Based on Weissberg et al. (2015), the SEL competences can be defined as: the ability to recognize one's emotions and thoughts and their influence on behavior (self-awareness); the ability to regulate one's emotions, thoughts, and behaviors in different situations (self-management); the ability to take the perspective of and empathize with others from diverse cultures and to understand social and ethical norms for behavior (social awareness); the ability to establish and keep healthy and rewarding relationships (relationship skills); the ability to make constructive and respectful choices about personal behavior and social interactions based on ethical standards, safety concerns, social norms, taking in consideration the well-being of self and others (responsible decision making). All these skills, when put in practice, help to promote well-being of both teachers and students, and enable their flourishing in the classroom (Talvi and Lonka, 2019). Social and emotional learning is thus the corner stone for positive development.

With the help of these skills one is capable of will nurturing more collective and cooperative behaviors, as well as decreasing behavior and communication problems, emotional tension, and developing effective problem solving, self-discipline, impulse control, and emotion management (Greenberg et al., 2003). SEL competences allow children to calm themselves when angry, make friends, resolve conflicts respectfully, and make ethical and safe choices (O'Brien and Resnik, 2009) and may empower individuals to become more responsible and empathic, promoting a dynamic participation in society and citizenship (Lemerise and Arsenio, 2000). SEL is a critical component of the educational experience that leads to improvements in student behavior, reductions in classroom disruption, and greater academic achievement. It does so by going beyond traditional academic skills by teaching students how to resolve conflicts, handle emotions, empathize, and make responsible decisions (Elias et al., 1997; Greenberg et al., 2003).

SEL is a complex process with which children and adults acquire and utilize skills to interact with oneself and others in a constructive and confident manner. These critical competencies of SEL are necessary for maintaining successful relationships with others, gaining meaningful employment, routing daily life skills, and problem-solving issues that arise in life, particularly as one move toward adulthood and greater self-sufficiency and autonomy. Interventions designed to teach constructive interaction skills as well as social and emotional skills to teachers aim at teachers' greater ability to both teach the same skills to their students and act as an example in different kinds of interactional situations in the classroom. Consequently, as teachers' gain more constructive ways of handling conflicts and challenging behavior the amount of problem behavior in the classroom diminishes (Elias et al., 1997; Greenberg et al., 2003).

### Teachers' Professional Development in SEL

With the view to developing a comprehensive model for the development and assessment of SEL skills, the goal of the Learning To Be-project was to develop and examine a set of innovative assessment methodologies in practice by conducting a number of field trials in schools in five partner countries. Further, evaluating the outcome of these assessment practices on the development of students' SEL skills and other learning outcomes (involvement in the community, motivation to learn etc.) was one of the core goals.

Research on teachers' SEL is still relatively scarce (Talvio, 2014). The lack of this research has been explained by the hypotheses that the development of teachers' skills is part of the tacit knowledge of the teaching profession (Elliott et al., 2011), or, that teachers' SEL develops as part of their role (Jennings and Greenberg, 2009). If it is suggested that teacher's SEL is best learned as part of their teaching practice, it follows that such knowledge may not be easily recognized or transmitted.

Some studies have investigated the benefits of SEL to teachers. Jennings and Greenberg (2009) found that teachers needed to spend less time on classroom management when SEL was effectively implemented in the classroom. Collie et al. (2012) found that beliefs about teachers' comfort in implementing SEL in the school settings results to the teacher higher levels of efficacy and personal accomplishment at the end of 1 year compared to the comparison sample. In addition, positive effects of the SEL workshops on school climate, student behaviors, and conflict resolution strategies have been reported (Collie et al., 2012; Gol-Guven, 2017). In addition, an Austrian longitudinal study found reduced bullying and fighting among the pupils whose teachers had participated in the training on SEL compared to the control group (Matischek-Jauk et al., 2018). The same study also found that the higher the implementation level of SEL, the more positive effects were found.

In order to succeed not only in promoting SEL in classrooms but also, in transferring the skills to the students, teacher's knowledge of the content taught and how to apply it is important. The extent of how faithfully the principles and activities are replicated, how much of the content is delivered, and how effectively the students' other studies and background are considered, are dependent on instructor's competence (Talvio et al., 2013, 2015). Accordingly, teachers' own development of SEL is crucial in the successful implementation process (Peeters et al., 2014). Studies on teachers' development during the SEL intervention indicated that teachers' knowledge and SEL skills increased in the intervention group (Talvio et al., 2019). Teachers learned to develop their social interaction skills, such as expressing their feelings in constructive ways. In addition, their readiness to use skills increased, non-desired ways of interacting decreased, and the teachers started thinking about how to support their students' autonomy (Talvio et al., 2013). The decrease in non-desired ways of interacting included avoiding blaming or reproaching students and withdrawing from distributing punishments or rewards. Hence, teachers benefit SEL both directly and indirectly; when they learn the skills themselves and when they teach the skills to their students. It is therefore important to investigate the benefits of an intervention that aims at improving teacher's social and emotional skills on students' SEL.

### Students' Social and Emotional Learning

Previous studies indicate that SEL increases students' chances of success in school and later life (Clarke et al., 2015; Weissberg et al., 2015). Elias et al. (1997) suggested that socio-emotional competence helps pupils to recognize and regulate their emotions effectively, communicate better with their peers and adults and form healthy and warm relationships with them. SEL is helpful also in meeting personal needs, setting realistic goals and making responsible decisions, all important elements for school motivation and school engagement (Greenberg and Jonas, 2003; Zins et al., 2004; Zins and Elias, 2007; O'Brien and Resnik, 2009).

However, the information about the effectiveness of the interventions is limited, as these interventions did not include a comparison group. Therefore, the benefits cannot be explained only because of the interventions (Corcoran et al., 2018). The meta-analysis of Taylor et al. (2017) was an exception focusing on SEL intervention studies with comparison conditions in school settings. It revealed statistically significant benefits of SEL for students including improved social and emotional skills and attitudes toward self, others, and school. In addition, SEL promoted pupils' prosocial behavioral and i.e., reduced conduct and internalizing problems. Positive effects on academic performance were found too.

Another problem according to Corcoran et al. (2018) is that there have been several reviews on the area of the benefits on SEL, but very few of them focus exclusively on SEL interventions. Most of them focuses, for example, on reducing bullying and victimization (Farrington and Ttofi, 2009), investigating the benefits of the use of mindfulness in the classroom (Maynard et al., 2015) or reducing problem behaviors and delinquency (Piquero et al., 2010). In addition to these, research can be found about the role of gender in problem behavior and competence (Forehand et al., 1991) and about using SEL framework in the selection of prevention programs that address health, substance abuse, violence prevention, sexuality, character, and social skills (Payton et al., 2000). Reducing student problem behavior remains one of the leading concerns for school staff, as disruptive and aggressive behavior interferes with student achievement and even the school climate. Research (Spaulding et al., 2010) shows that problem behavior is most likely to be generated from classrooms and more likely to be related to peer-directed problem behavior in elementary schools, student-adult interactions in middle schools, and tardiness and truancy in high schools. Problem behavior in the present study is defined as *behavior that violates definitions of appropriate conduct and norms shared by the members of a social system* (Jessor, 1982). For students, it typically consists of behavioral patterns that are correlated with adverse social, psychological and physical consequences, such as substance abuse and physiological or psychological violence (Georges, 2009). Given the behavioral expectations in the classroom (e.g., sustained attention on task, motivation for individual studying and participating in cooperative or collaborative group work, etc.), bullying, substance abuse and truancy place children and adolescents at risk of not being able to live up to these expectations. Furthermore, continuing or repetitive breaking of the school rules brings both negative attention and feedback placing these children under the risk of negative development caused by a negative perception of one self and ones' abilities.

### Aims

On one hand, national curricula frameworks all over Europe underline the importance of social and emotional skills in education. On the other hand, there has been a lack of awareness on how to assess social and emotional skills and how to integrate assessment strategies of these skills into the existing education practices. This project aimed at producing an intervention providing teachers' with skills to teach and assess social and emotional learning in the classroom.

The aim of this study was to look at the students' development of their SEL *per se* as a result of the effect of the intervention provided to their teachers in the school context. The design included two age groups: pre-puberty aged 8–11 year-old students and 12–15 year-old adolescents as both intervention groups and comparison groups to capture the effect of the SEL intervention as well as attempting to capture the most fruitful timing in terms of the age of the participating students in five European countries. Another aim was to look if there was a negative development in students' deviant behavior.

In this report the research questions are:

Does the *SEL competencies assessment practices* intervention developed in the Learning to Be-project have a positive desirable development in terms of:

(1) Teachers' perceived readiness to implement SEL learning; This was operationalized by using the following variables: teachers' *perceptions of the importance* of teaching SEL and teachers' *perceptions of their competence* in teaching SEL
(2) Students' SEL competencies and is there a difference between the two participating age groups (the group of 8–11 and 12–15)?
(3) Reducing students' problem behavior—are the results different in the two age groups?

In this study also significances under 0.10 (10%) are presented in an attempt to capture the possible trends of development. However, only statistically significant results are discussed further.

## Methods

### Context of the Study

This study is about an experimental project called “Learning to Be: Development of Practices and Methodologies for Assessing Social, Emotional and Health Skills within Education Systems” in the framework of Erasmus+ KA3 programme (582955-EPP-1-2016-2-LT-EPPKA3-PI-POLICY). The project brought together education authorities, teaching practitioners and researchers from seven European countries: Finland, Italy Latvia, Lithuania, Portugal, Slovenia, and Spain.

The Finnish research group was responsible for the independent evaluation. The interventions were carried out in Italy, Latvia, Lithuania, Slovenia, and Spain. Researchers from these countries helped to understand the cultural context, cross-translated the questionnaires and implemented the interventions. The contribution of Portugal was in participating in the design of the Toolkit providing the assessment tools used in the interventions in schools.

An intervention for teachers in primary and lower secondary schools was designed in an attempt to make SEL more visible in schools by proposing methods for teachers to assess students' progress and support their further learning. The intervention included a Toolkit (Agliati et al., 2020) for teachers. The Toolkit was created in co-operation with the participating institutes from the five European countries involved in the field trials. The manual for the Toolkit included: a theoretical introduction to SEL providing guidelines for consistent practice, descriptions of teaching methods, assessment tools for teachers and students and SEL learning standards that present learning objectives for two age groups of students. The Toolkit was translated to all five target languages, namely Italian, Latvian, Lithuanian, Slovenian, and Spanish languages. An additional translation to English language was also made.

### Interventions at Schools

The toolkit was created as training material for the teachers who participated in the intervention. The length of the training provided for teachers in the intervention group was 16 h. The 2-day training programme was based on an experiential SEL methodology, modeling (practicing) the same methods for classroom learning, community involvement and assessment of SEL skills that teachers were expected to transfer to their school life. The first part of the workshop focused on understanding SEL and discussing its implementation at school. The next 10-h programme was aimed at teachers, and focused on the practical parts of the Toolkit: learning methods to support SEL, strategies for creating a supportive social environment at school and formative assessment of SE skills. After the training, the experimental schools piloted the Toolkit in the classroom. The agreed duration of the pilot (intervention) continued for 5 months in each experimental school. Additionally, the country coordinators arranged an additional 9 h of monitoring in an attempt to reinforce the acquisition of skills, practices and knowledge gathered during the training as well as to gather data for qualitative research purposes. The length of the training for principals and head masters varied from 10 to 16 h. Despite the variation in the length of the training for the administration, both the pre-tests and the post-tests were conducted at approximately the same time in each of the participating countries, The pre-tests were carried out in September and October in 2018, and the posttests were carried out in May and June in 2019.

### Participants

Data were collected from teachers and their students in five participating countries (Italy, Latvia, Lithuania, Slovenia, and Spain). The randomly selected schools for both intervention and comparison groups in each country were designated to represent typical schools in each target country, including both urban and rural schools having not participated in SEL interventions previously. Overall, the sample of the study intended to be as representative as possible aiming at exploring the average change in ordinary schools and in ordinary learning groups in each country. Tables 1, 2 show the distribution of teachers and students per country.

**Table 1:** Number of teachers according to gender in all participating countries and in total.

	**Italy**				**Latvia**				**Lithuania**				**Slovenia**				**Spain**				**Total**			
	***n*** = **84**				***n*** = **92**				***n*** = **105**				***n*** = **44**				***n*** = **84**				***n*** = **409**			
**G**	**Int**		**Comp**		**Int**		**Comp**		**Int**		**Comp**		**Int**		**Comp**		**Int**		**Comp**		**Int**		**Comp**	
	***n***	**%**	***n***	**%**	***n***	**%**	***n***	**%**	***n***	**%**	***n***	**%**	***n***	**%**	***n***	**%**	***n***	**%**	***n***	**%**	***n***	**%**	***n***	**%**
F	38	84	37	95	57	93	27	87	61	90	37	100	26	79	10	91	29	67	28	68	211	87	139	87.4
M	0	0	2	5.1	3	4.9	2	6.5	5	7.4	0	0	4	12	1	9.1	13	30	13	32	25	10	18	11.3
D	6	16	0	0	1	1.6	2	6.5	2	2.9	0	0	3	9.1	0	0	1	2.3	0	0	16	6.6	2	1.3
T	45	100	39	100	61	100	31	100	68	100	37	100	33	100	11	100	43	100	41	100	243	100	159	100

*The number of teachers in each country consists of those who participated in both pre-test and post-test*.

*G, gender; F, female; M, male; D, do not wish to tell; T, total*.

**Table 2:** Characteristics of the teachers of the intervention and comparison groups.

	**Intervention group**	**Comparison group**
Teacher position in the community	(*n* = 675)	(*n* = 287)
Subject matter teachers	331 (48.3%)	199 (50.8%)
Class teachers	273 (39.9%)	145 (37%)
Special needs teachers	16 (2.3%)	13 (3.3%)
Other	55 (8.0%)	30 (7.7%)
Missing	10 (1.5%)	5 (1.7%)
Gender	(*n* = 674)	(*n* = 387)
Female	580 (84.7%)	339 (86.5%)
Male	70 (10.2%)	39 (9.9%)
Did not tell	24 (3.5%)	9 (2.3%)
Missing	11 (1.6%)	5 (1.3%)
Average age	(*n* = 642)	(*n* = 374)
	46.8 years	45.7 years
Average experience in years	21.7 years	21.0 years
Minimum	0	0
Maximum	47 years	58 years

#### Teachers

In order to be eligible for participation in the evaluation part of the project, teachers participating in the present study had to meet the following criteria: not participate in previous SEL training and work in either elementary or secondary school (or another national equivalent). In addition the intervention group teachers needed to participate in the training whereas the comparison group teachers did not participate in any SEL training during the time of the study. The comparison groups should be as similar as possible with the intervention groups.

Total research sample of teachers consisted of an *intervention group* (*n* = 243) who participated in the intervention and a *comparison* group (*n* = 159) who did not take part in the intervention. Table 1 shows that the largest intervention group of teachers was from Latvia (*n* = 61) and the smallest from Slovenia (*n* = 33). The largest comparison group of teachers was from Spain (*n* = 41) and smallest from Slovenia (*n* = 11). Participants in both intervention and comparison groups were selected by the national research coordinator from randomly selected schools. Educators had to be schoolteachers or other personnel directly involved in educational work with children in the school community e.g., social workers/educators, school psychologists, educators of non-formal learning programs (art/sports groups, community, and youth organizations etc.). Despite the possibility for other personnel working in the field of education being eligible to participate, all the participants were teachers.

The difference in background information were compared to report the possible differences between the intervention and the comparison groups as well as between genders in each country by using Chi-square test. Table 1 shows the total number of teachers who participated in both the pre- and the post-test (*n* = 402) and also that there was a significant difference in the number of male and female teachers.

The characteristics of the participants (e.g., type of teacher, gender, age, and average experience in years) were quite similar in the intervention and comparison groups. For detailed information see Table 2.

#### Students

The students were grouped into two age groups: 8–11-years old (pre puberty) and 12–15-years old (adolescents). Table 3 shows the exact numbers of students and their gender in both age groups in each country. Students whose teachers had participated in the intervention belonged to the intervention group. Those students whose teachers had not participated in the intervention group belonged to the comparison group. In an attempt to ensure all members of the learning groups were provided the possibility for participating in the research, additional translations were made for minority language groups in some of the participating countries.

**Table 3:** Students' distribution as a function of age and gender across countries.

**Country**	**Age group**																												
**Italy** (***n*** **= 1,826)**		**Only pre-test (***n*** = 305)**														**Both pre-test and post-test (***n*** = 1,521)**													
		**int (***n*** = 202)**							**com (***n*** = 103)**							**int (***n*** = 986)**							**com (***n*** = 535)**						
		**g**	**%**	**b**	**%**	**d**	**%**	**tot**	**g**	**%**	**b**	**%**	**d**	**%**	**tot**	**g**	**%**	**b**	**%**	**d**	**%**	**tot**	**g**	**%**	**b**	**%**	**d**	**%**	**tot**
	8–11	47	40	63	53	7	0	117	15	31	28	57	6	12	49	234	50	208	45	25	5	467	145	54	117	43	9	3	271
	12–15	40	47	38	45	7	8	85	15	28	38	70	1	2	54	186	36	325	63	8	2	519	117	44	138	52	9	3	264
**Latvia** (***n*** **= 1,820)**		**Only pre-test (***n*** = 624)**														**Both pre-test and post-test (***n*** = 1,521)**													
		**int (***n*** = 327)**							**com (***n*** = 297)**							**int (***n*** = 704)**							**com (***n*** = 492)**						
		**g**	**%**	**b**	**%**	**d**	**%**	**tot**	**g**	**%**	**b**	**%**	**d**	**%**	**tot**	**g**	**%**	**b**	**%**	**d**	**%**	**tot**	**g**	**%**	**b**	**%**	**d**	**%**	**tot**
	8–11	53	41	73	57	3	2	129	45	43	56	54	3	3	104	187	50	172	46	13	3	372	126	55	100	43	4	2	230
	12–15	83	42	106	54	9	5	198	79	41	106	55	8	4	193	183	55	134	40	15	5	332	131	50	116	44	15	6	262
**Lithuania** (***n*** **= 1,216)**		**Only pre-test (***n*** = 467)**														**Both pre-test and post-test (***n*** = 749)**													
		**int (***n*** = 183)**							**com (***n*** = 284)**							**int (***n*** = 379)**							**com (***n*** = 370)**						
		**g**	**%**	**b**	**%**	**d**	**%**	**tot**	**g**	**%**	**b**	**%**	**d**	**%**	**tot**	**g**	**%**	**b**	**%**	**d**	**%**	**tot**	**g**	**%**	**b**	**%**	**d**	**%**	**tot**
	8–11	42	45	50	53	2	2	94	82	54	62	41	7	5	151	85	49	82	47	8	5	175	91	47	94	48	9	5	194
	12–15	42	47	45	51	2	2	89	70	53	59	44	4	3	133	121	59	76	37	7	3	204	97	55	76	43	3	2	176
**Slovenia** (***n*** **= 567)**		**Only pre-test (***n*** = 271)**														**Both pre-test and post-test (***n*** = 296)**													
		**int (***n*** = 93)**							**com (***n*** = 178)**							**int (***n*** = 203)**							**com (***n*** = 93)**						
		**g**	**%**	**b**	**%**	**d**	**%**	**tot**	**g**	**%**	**b**	**%**	**d**	**%**	**tot**	**g**	**%**	**b**	**%**	**d**	**%**	**tot**	**g**	**%**	**b**	**%**	**d**	**%**	**tot**
	8–11	13	37	18	51	4	11	35	45	38	54	45	#	17	119	83	53	70	44	5	3	158	18	34	25	47	10	19	53
	12–15	37	64	18	31	3	5	58	26	44	26	44	7	12	59	23	51	22	49	0	0	45	24	60	16	40	0	0	40
**Spain** (***n*** **= 1,379)**		**Only pre-test (***n*** = 859)**														**Both pre-test and post-test (***n*** = 520)**													
		**int (***n*** = 505)**							**com (***n*** = 354)**							**int (***n*** = 280)**							**com (***n*** = 240)**						
		**g**	**%**	**b**	**%**	**d**	**%**	**tot**	**g**	**%**	**b**	**%**	**d**	**%**	**tot**	**g**	**%**	**b**	**%**	**d**	**%**	**tot**	**g**	**%**	**b**	**%**	**d**	**%**	**tot**
	8–11	113	46	123	50	10	4	246	63	49	59	46	7	5	129	83	51	72	44	8	5	163	87	56	59	38	8	5	154
	12–15	118	46	132	51	9	3	259	82	36	136	60	7	3	225	37	32	72	62	8	7	117	37	43	39	45	10	12	86

*g, girls; b, boys; d, do not wish to tell*.

#### Students' Intervention Group

Total research sample of students' intervention group consisted of 203–986 students depending on the country. Altogether 2,552 students (see Table 3). In order to be eligible for participation in the evaluation part of the project participants had to meet the following criteria: age between 9–11 (group 1) and 13–15 years old (group 2). These age groups were selected based on a fact that generally education is compulsory until 16 years old. The *Students' comparison group* consisted of 93–492 students depending on the participating country, altogether 1,730 students (see Table 3). In order to be eligible for participation in the evaluation part of the project the participants had to meet the following criteria: age between 9–11 and 13–15 years old. In this group, there were also students who reported to be 8 or 12 years old and due to their upcoming birthday, the age groups were widened to 8–11 and 12–15.

Table 3 shows the number of participating students in both pre-test and post-test. This study focuses on the group of students who attended both pre- and post-tests because in this group of students it is possible to investigate their development in SEL.

### Data Collection

Data from teachers and students were collected before (pre-test) the intervention. It was collected from both intervention and comparison groups at the beginning of the school year in September. Participants filling in the electric questionnaire were informed that their information and responses would remain anonymous. Participants were also informed about the possibility of withdrawing their responses from this study at any time without warning or explanation in advance. Students' parents were asked for their informed consent by the school for their right not to let their child to participate in the study. Post-test data from teachers and their students in both intervention and comparison groups were collected right after the intervention at the end of the school year. The questionnaire used to collect the data in both pre- and post-tests was on an electronic platform called Survey Gizmo. A paper-version of the same questionnaire was available in cases where it was impossible to use the electronic version, for example, due to a poor internet connection or other problem with the electronic system. These paper versions were added manually to the electronic file by the country coordinators.

Participants who did not give the consent, or saved empty, nearly empty or clearly implausible (for example only answering maximum or minimum values) answers were removed from the database.

### Ethical Considerations

Ethical review board in the humanities and social and behavioral sciences of the University of Helsinki was requested to give a review for the project.

GDPR regulations were taken into account in protecting the privacy of the participants who were instructed to create a 6-digit code, which then was replaced in Helsinki by a participant number. As the collected data concerning the participating countries was provided to the partner researchers, all data that might enable the identification of an individual participant (id-code, school name) was deleted and replaced by the participant number. The data file matching the participant numbers to the id-codes was saved in a separate file to enable the matching of pre- and post-test answers.

### Measures

Teachers and students completed a set of questionnaires covering their well-being, epistemic beliefs and other aspects that are beyond the scope of this study. For the scope of this study, we only focus on the questions about SEL competencies and problem behavior, because they were the target of the interventions. With regard to teachers' questionnaire 12 questions concerned their knowledge of SEL and 7 questions concerned their perceived skills in implementing SEL in their classrooms. All questions concerning SEL as well as implementing SEL were presented as the last questions in the questionnaire, right before questions concerning background information.

With regard to students' questionnaire, they were asked to answer 25 questions concerning SEL, bullying, health and well-being, and self-esteem. A pilot test was conducted in an attempt to verify that the questions were easy to comprehend even for the younger group of students. As no difficulties were encountered, the questionnaires for both age groups of students (8–11- and 12–15-year-olds) were the same. However, the younger students were provided with more time and opportunities for asking questions while filling in the questionnaire. Out of the 78 questions 25 concerned SEL and they were located at the end of the questionnaire right before questions concerning background information. The other questions concerned bullying, health and well-being and self-esteem.

Teachers' perceived readiness to implement SEL learning was measured using scale (for scale validation see Talvio et al., 2016) that consisted of two components: teachers' *perceptions of the importance* of teaching SEL and teachers' *perceptions of their competence* in teaching SEL. Perceptions of the importance of teaching SEL were measured using 7 items that participants evaluated on seven-point Likert scale with response options ranging from “*not at all important*” (1) to “*very important*” (7). Examples of statements used to measure participants' perceptions of the importance of teaching SEL included “It is primarily the teacher's duty to create a classroom environment where all students feel valued” and “It is the teacher's duty to teach interactive skills such as listening and conversation skills.” Perceptions of competence were measured using seven items, that participants evaluated on seven-point Likert scale with response options ranging from “*strongly disagree”* (1) to “*strongly agree”* (7). Teachers' opinions of their competence was investigated using statements such as “*I am very skilled at creating a classroom environment where all students feel valued”* and “*I am very skilled at teaching interactive skills such as listening and conversation skills.”*

Students' SEL competencies were investigated using Social Emotional Competence Questionnaire (SECQ) (for scale validation see, Zhou and Ee, 2012), that consisted of 25 items and five components: self-awareness, social awareness, self-management, relationship management and responsible decision-making (Table 4). Participants evaluated the items on six-point Likert scale with response options ranging from “*Completely false”* (1) to “*Completely true”* (7). Self-awareness relates to skills in recognizing and identifying one's own emotions, strengths and weaknesses, and understanding how they affect one's behavior. Example of an item used to investigate self-awareness: “*I know what I am thinking and doing.”* Social awareness is the ability to understand other persons' feelings and accordingly respond to their feelings. This was measured for example with an item: “*If a friend is upset, I have a pretty good idea why.”* Self-management is the ability to manage one's own emotional experiences and impulses. This was measured for example with an item: “*I can control the way I feel when something bad happens.”* Relationship management refers to skills in building and maintaining relationships, conflict management and cooperation. This was measured for example with an item: “*I am tolerant of my friend's mistakes.”* Responsible decision-making is the ability to consider ethical and societal factors in making decisions. One of the items used to measure this was: “*When making decisions, I take into account the consequences of my actions.”*

**Table 4:** Items, variables and Cronbach's alpha and internal consistencies of the sum variables that measure students' social and emotional competence across all countries.

**Items**	**Sum variable's name**	**Cronbach's alpha**
1. I know what I am thinking and doing.	S1	0.72
2. I understand why I do what I do.	(Self-awareness)	
3. I understand my moods and feelings.		
5. I can read people's faces when they are angry.	S2	0.80
6. I recognize how people feel by looking at their facial expressions.	(Social awareness)	
7. It is easy for me to understand why people feel the way they do.		
8. If someone is sad, angry or happy, I believe I know what they are thinking.		
9. I understand why people react the way they do.		
10. If a friend is upset, I have a pretty good idea why.		
11. I can stay calm in stressful situations.	S3	0.80
12. I stay calm and overcome anxiety in new or changing situations.	(Self-management)	
13. I stay calm when things go wrong.		
14. I can control the way I feel when something bad happens.		
16. I will always apologize when I hurt my friend unintentionally.	R1	0.76
17. I always try and comfort my friends when they are sad.	Relationship management)	
18. I try not to criticize my friend when we quarrel.		
19. I am tolerant of my friend's mistakes.		
20. I stand up for myself without putting others down.		
21. When making decisions, I take into account the consequences of my actions.	R2	0.84
22. I try to make choices that have the most positive outcomes expected.	(Responsible	
23. I weigh the strengths of the situation before deciding what I will do.	decision-making)	
24. If I make a recommendation, I think about the criteria behind my recommendation.		
25. I consider the strengths and weaknesses of the strategy before deciding to use it.		

Table 4 shows the content of the student questionnaire concerning SEL and the number of questions regarding each component.

The internal consistency of the students' SEL scales varied between 0.72 and 0.84 (Cronbach's alpha) showing moderate to good internal consistency. The results reported were based on the sum scores of the pre-test.

In this study, students' problem behavior was defined as bullying, truancy and substance abuse. These items were measured by using a three-point or five-point Likert scale. Bullying and participating in physical fights were measured by asking “*How many times were you involved in bullying during the last month?* and “*How many times were you involved in a physical fight during the last month?”* using a five-point Likert scale ranging from response options “*I have not bullied at school during the last month”* (1) to “*I have bullied at school once a week”* (5) and *I have not been in physical fights during the last month* (1) to *four times or more* (5). Truancy was measured by asking “*Have you been absent due to skipping on purpose during the last month?”* and with the response options ranging from “*None”* (1) to “*More than 5 days”* (5). The frequency of possible substance abuse was measured by using a three-point Likert scale with the response options ranging from “*No”* (1) to “*Now and then”* (3). The likelihood of yielding to social pressure was measured with a question “*If one of your best friends was to offer you any of these, would you use it?”* separately for alcohol, tobacco and drugs and the response options ranging from “*I would not know what it is”* (1) to “*Certainly yes”* (5).

The sum variable “Problem behavior” was constructed by forming a sum score of these items and scaling them to start from zero. The internal consistency of the students' problem behavior-scale scale was 0.68 in the pre-test and 0.70 in the post test (Cronbach's alpha) showing moderate to good internal consistency. The results reported are based on the sum scores of the post test.

### Statistical Procedures

Statistical differences between the scores of the pre-test and the post-test were examined with repeated measures ANOVA (GLM). SPSS 25 was used in the analyses. The mean sum scores were conducted from the multi-item measures and used these as variables in further analyses. Repeated measures ANOVA tested the “time^*^group” and “time^*^group^*^age group” interaction examining the effect of the intervention with regards to mean change over time across groups in the variables. The analyses were conducted separately for each country and to all countries combined.

Furthermore, the difference between the number of representatives in gender groups was tested as well as the possible change between pre- and post-tests between and within the age groups. These were statistically controlled for different age groups in evaluating the effect of the intervention. The possible effect of students' age and gender as background variables were thus taken into account.

## Results

### Results Country by Country

In order to answer the first research question, “Did the *SEL competences assessment practices intervention* result to a positive development on teachers' perceived readiness to implement SEL learning?” Table 5 shows that, all teachers, regardless of being in the intervention or comparison group, scored very high in their perceived *importance* of social and emotional learning both in pre- and post-tests. The lowest mean value was in Lithuania and the highest was in Italy. Concerning their perceived SEL *competence*, all teachers scored in the pre-test between 5.3 and 5.5. The lowest mean value was in Latvia and the highest in Lithuania. Repeated measures ANOVA (GLM) was used to test the gain scores between and within (pre- and post-tests) groups examining the effect of the intervention with regards to mean change over time across the groups. The analyses were conducted separately for each country and to all countries combined. The investigations of the data revealed that no statistically significant changes were found in the analysis concerning teachers' SEL competencies.

**Table 5:** Teachers perceived importance and competence in SEL.

		**Intervention**				**Comparison**						
		**Pre**		**Post**		**Pre**		**Post**				
		***n***	***M (SD)***	***n***	***M (SD)***	***n***	***M (SD)***	***n***	***M (SD)***	***F (df)***	***p***	**Partial Eta Sq**
Italy	Im	38	6.6 (0.37)	38	6.6 (0.45)	37	6.3 (0.52)	37	6.3 (0.67)	0.187 (1, 75)	0.67	0.003
	Co	38	5.3 (0.91)	38	5.5 (0.74)	37	5.3 (0.74)	37	5.3 (0.76)	1.09 (1, 74)	0.30	0.15
Latvia	Im	62	6.2 (0.65)	62	6.0 (0.65)	30	6.0 (0.59)	30	6.0 (0.46)	1.96 (1, 91)	0.17	0.021
	Co	62	5.3 (0.62)	62	5.3 (0.62)	30	5.4 (0.9)	30	5.3 (0.50)	0.23 (1, 91)	0.63	0.003
Lithuania	Im	66	6.1 (0.60)	66	6.3 (0.49)	35	6.0 (0.79)	35	6.1 (0.74)	0.93 (1, 100)	0.34	0.009
	Co	66	5.5 (0.56)	66	5.6 (0.56)	35	5.6 (0.78)	35	5.5 (0.70)	0.95 (1, 100)	0.33	0.010
Slovenia	Im	41	6.3 (0.67)	41	6.2 (0.78)	41	6.3 (0.48)	41	6.2 (0.65)	2.05 (1, 43)	0.16	0.046
	Co	41	5.4 (0.79)	41	5.6 (0.87)	41	5.3 (0.78)	41	5.4 (0.84)	1.21 (1, 42)	0.28	0.029
Spain	Im	33	6.4 (0.45)	33	6.6 (0.49)	11	6.5 (0.6)	11	6.4 (0.85)	0.21 (1, 81)	0.65	0.003
	Co	33	4.7 (0.9)	33	4.5 (0.8)	10	5.7 (0.90)	10	6.0 (0.66)	0.43 (1, 81)	0.51	0.005

*Im, perceived importance; Co, perceived competence*.

The second and third research questions were: “Is there a difference between the two participating age groups (the age group of 8–11-years old and the age group of 12–15-years old) in terms of SEL competencies and in the amount of students' problem behavior?”

First, we studied the possible change in the five core components of SEL in within the intervention and comparison groups as well as between these two groups in both of the age groups between pre- and post-tests in each of the participating countries. In the second phase we studied the possible change in the five elements of SEL as well as the possible change in Problem behavior within intervention and comparison groups as well as within both age groups between pre- and post-tests and between the intervention and the comparison groups with all the participants from all countries combined.

Table 6 describes the number of participants, mean values, standard deviations in pre- and post-tests in both intervention and comparison groups. Interaction effects of all variables of SEL are provided here country by country. The scores are presented for two age groups individually. As can be seen, significant changes took place or there was a significant interaction between age, only after taking the variance between the age groups within both the intervention and comparison groups and between measurement points (pre-test and post-test) into account. The only interaction effects approaching significance were observed between the intervention and the comparison groups in social awareness among the younger age group in Latvia, and in self-management in Lithuania.

**Table 6:** Number of participants, mean values, standard deviations and interaction effects of all variables of SEL country by country in alphabetical order.

**Country**	**Age group**	**Variable**	**Intervention**				**Comparison**									
			**Pre**		**Post**		**Pre**		**Post**		**T × IntCom**			**T × IntCom × Age**		
			***n***	***M (SD)***	***n***	***M (SD)***	***n***	***M (SD)***	***n***	***M (SD)***	***F (df)***	***p***	***d***	***F (df)***	***p***	***d***
Italy	8–11	S1	584	4.8 (0.7)	449	4.6 (0.6)	320	4.7 (0.7)	310	4.7 (0.6)	1.578 (1, 1,350)	0.21	0.001	1.656 (1, 1,350)	0.20	0.001
		S2	584	4.1 (0.9)	449	3.9 (0.8)	320	4.0 (1.0)	310	4.1 (0.8)	0.011 (1, 1,351)	0.91	0	4.087 (1, 1,351)	0.04^*^	0.003
		S3	584	3.8 (1.1)	449	3.4 (1.0)	320	3.7 (1.1)	310	3.4 (1.0)	2.227 (1, 1,349)	0.14	0.002	0.165 (1, 1,349)	0.69	0
		R1	583	4.7 (0.9)	449	4.5 (0.8)	319	4.5 (0.9)	310	4.5 (0.8)	0.127 (1, 1,346)	0.72	0	0.333 (1, 1,346)	0.56	0
		R2	583	4.8 (0.9)	449	4.5 (0.8)	318	4.7 (0.9)	309	4.5 (0.7)	1.212 (1, 1,345)	0.27	0.001	3.241 (1, 1,345)	0.07	0.002
	12–15	S1	465	4.8 (0.7)	370	4.7 (0.6)	263	4.8 (0.7)	262	4.7 (0.6)						
		S2	465	4.0 (1.0)	370	4.0 (0.8)	263	4.1 (1.0)	262	4.0 (0.8)						
		S3	465	3.7 (1.1)	370	3.4 (1.1)	263	3.8 (1.1)	261	3.5 (1.0)						
		R1	464	4.7 (0.8)	370	4.5 (0.8)	262	4.7 (0.8)	261	4.4 (0.8)						
		R2	465	4.7 (0.9)	370	4.5 (0.8)	262	4.7 (0.8)	261	4.5 (0.8)						
Latvia	8–11	S1	501	4.5 (1.0)	530	4.4 (0.9)	335	4.6 (1.0)	434	4.6 (0.7)	0.534 (1, 1,187)	0.47	0	0.153 (1, 1,187)	0.70	0
		S2	498	3.8 (1.1)	524	3.7 (0.9)	333	3.8 (1.1)	431	3.9 (0.9)	3.395 (1, 1,178)	0.07	0.003	0.299 (1, 1,178)	0.58	0
		S3	493	3.9 (1.0)	523	3.7 (1.0)	328	4.1 (1.0)	431	3.9 (0.9)	2.145 (1, 1,168)	0.43	0.001	1.981 (1, 1,168)	0.16	0.002
		R1	494	4.5 (1.0)	515	4.3 (0.9)	328	4.6 (1.0)	428	4.5 (0.8)	0.626 (1, 1,170)	0.43	0	0.402 (1, 1,170)	0.53	0
		R2	496	4.5 (0.9)	515	4.1 (0.9)	327	4.6 (1.0)	425	4.3 (0.9)	0.041 (1, 1,165)	0.84	0	0.015 (1, 1,165)	0.9	0
	12–15	S1	370	4.5 (1.0)	330	4.5 (0.8)	230	4.7 (0.8)	263	4.6 (0.8)						
		S2	367	3.9 (1.1)	327	3.9 (0.9)	230	3.9 (0.9)	263	4.0 (0.9)						
		S3	367	4.0 (1.0)	325	3.8 (1.0)	230	4.1 (1.0)	261	3.9 (1.0)						
		R1	368	4.5 (0.9)	327	4.4 (0.8)	229	4.7 (0.9)	262	4.4 (0.8)						
		R2	367	4.4 (1.0)	325	4.2 (0.8)	230	4.6 (1.0)	262	4.3 (0.9)						
Lithuania	9–11	S1	269	4.6 (1.2)	293	4.8 (0.9)	346	4.7 (1.0)	309	4.9 (0.8)	1.549 (1, 735)	0.21	0.002	3.682 (1, 735)	0.06	0.005
		S2	268	4.2 (1.3)	293	4.3 (1.1)	346	4.4 (1.0)	308	4.2 (1.0)	0.098 (1, 733)	0.75	0.002	1.977 (1, 733)	0.16	0.003
		S3	265	4.0 (1.3)	294	4.3 (1.0)	343	4.3 (1.1)	305	4.0 (0.9)	3.13 (1, 726)	0.08	0.004	0.424 (1, 726)	0.51	0.001
		R1	268	4.4 (1.2)	294	4.9 (0.9)	344	4.8 (0.9)	309	4.6 (0.8)	0.134 (1, 730)	0.71	0	1.868 (1, 730)	0.17	0.003
		R2	268	4.5 (1.1)	294	4.8 (0.9)	344	4.6 (1.1)	308	4.5 (0.9)	0.109 (1, 731)	0.74	0	2.294 (1, 731)	0.13	0.003
	12–15	S1	173	4.7 (1.0)	203	5.0 (0.8)	191	4.7 (1.0)	173	4.8 (0.8)						
		S2	173	4.3 (1.1)	203	4.5 (1.0)	189	4.2 (1.2)	173	4.3 (1.0)						
		S3	172	4.0 (1.2)	203	4.5 (1.0)	186	4.0 (1.2)	173	4.0 (1.0)						
		R1	171	4.5 (1.1)	202	5.0 (0.9)	188	4.7 (1.1)	173	4.7 (0.8)						
		R2	172	4.4 (1.2)	203	4.8 (0.9)	188	4.5 (1.1)	172	4.6 (0.9)						
Slovenia	8–11	S1	193	4.9 (0.8)	126	5.0 (0.8)	168	5.0 (0.8)	98	5.0 (0.7)	0.002 (1, 287)	1.0	0	0.009 (1, 287)	0.9	0
		S2	193	4.2 (1.1)	126	4.3 (0.8)	166	4.1 (1.2)	98	4.1 (0.8)	1.846 (1, 285)	0.18	0.006	6.781 (1, 285)	0.01^*^	0.023
		S3	193	4.3 (0.8)	126	4.2 (1.0)	163	4.3 (1.0)	96	4.1 (0.9)	0.286 (1, 282)	0.59	0.001	0.703 (1, 282)	0.40	0.002
		R1	193	4.9 (0.8)	126	4.9 (0.7)	171	4.6 (1.0)	98	4.9 (0.7)	0.314 (1, 289)	0.58	0.001	1.361 (1, 289)	0.24	0.005
		R2	193	4.8 (0.8)	124	4.7 (0.8)	171	4.5 (1.0)	98	4.7 (0.7)	0.005 (1, 289)	0.95	0	0.086 (1, 289)	0.77	0
	12–15	S1	158	5.2 (0.8)	45	4.9 (0.9)	53	5.0 (0.7)	40	5.0 (0.8)						
		S2	157	4.3 (1.1)	45	4.4 (1.0)	53	4.1 (1.0)	40	4.4 (0.9)						
		S3	157	4.3 (1.2)	45	4.1 (1.1)	53	4.4 (1.0)	40	4.0 (1.0)						
		R1	157	5.0 (0.8)	45	4.8 (0.8)	53	5.0 (0.8)	39	5.1 (0.8)						
		R2	157	4.9 (0.9)	45	4.5 (0.9)	53	4.7 (0.9)	39	4.6 (1.0)						
Spain	8–11	S1	409	5.2 (0.8)	346	5.0 (0.7)	282	5.1 (0.7)	416	5.0 (0.7)	0.349 (1, 576)	0.56	0.001	11.549 (1, 576)	0.001^*^	0.02
		S2	406	4.4 (1.1)	345	4.2 (0.9)	280	4.1 (1.1)	415	4.3 (0.9)	0.878 (1, 570)	0.34	0.003	0.009 (1, 570)	0.91	0
		S3	404	4.5 (1.1)	341	4.1 (1.0)	279	4.2 (1.1)	412	4.1 (1.1)	2.422 (1, 564)	0.12	0.004	0.142 (1, 564)	0.70	0
		R1	405	5.1 (0.8)	341	4.9 (0.8)	279	5.1 (0.9)	415	4.9 (0.8)	0.467 (1, 566)	0.50	0.001	3.039 (1, 566)	0.08	0.005
		R2	403	5.1 (0.9)	343	4.7 (0.9)	278	4.9 (0.9)	413	4.8 (0.9)	2.117 (1, 563)	0.15	0.004	5.114 (1, 563)	0.02^*^	0.009
	12–15	S1	162	5.4 (0.7)	84	4.7 (0.9)	152	5.1 (0.9)	182	4.9 (0.8)						
		S2	162	4.5 (1.2)	84	4.1 (0.8)	151	4.2 (1.0)	180	4.4 (0.8)						
		S3	159	4.5 (1.2)	84	3.7 (1.0)	151	4.3 (1.1)	179	4.0 (1.1)						
		R1	157	5.3 (0.7)	84	4.6 (0.8)	152	5.2 (0.8)	179	4.8 (0.9)						
		R2	157	5.3 (0.7)	84	4.4 (1.0)	152	5.0 (0.8)	177	4.7 (0.9)						

* *p < 0.05. S1, Self-awareness; S2, Social awareness; S3, Self-management; R1, Relationship management; R2, responsible decision making*.

As Table 6 shows, there were no significant differences between pre- and post-tests in any country, in terms of students' SEL in country by country comparisons. There were some almost significant (*p* = 0.07–0.08) trends in some of the variables studied: Social awareness improved in the Latvian student sample in both age groups as well as in Slovenian 8–11-year old student sample. There was also a non-significant positive change in the Lithuanian 8–11-year old student sample in both self-awareness and self-management as well as in self-awareness and relationship skills in the 8–11-year old Spanish student sample. Responsible decision making slightly improved in the age group of 12–15-year old students in the Italian sample, whereas the there was a negative change in the same variables in the 8–11-year old Italian student sample.

Table 6 also shows that there was a significant interaction between age, group and SEL skills in some countries: Some of these changes were negative indicating that the change in question was not desirable: Italian 8–11-year old students slightly decreased in their experienced social awareness and responsible decision making. Spanish 12–15-year old students slightly decreased in their self-awareness, relationship skills and responsible decision making during the intervention. There was also a negative development in the Slovenian 12–15-year old students' social awareness. These results did not reach statistical significance.

Statistical analysis revealed no statistically significant changes between these measurements, when the countries were looked at separately. Therefore, we combined the results of all countries.

### Results After Combining the Countries

During the second phase, the perceived SEL was studied across all five core components of SEL as well as Problem behavior with all the countries together. Table 7 shows the summary of the combined results.

**Table 7:** SEL and problem behavior in both age groups separately in pre- and post-tests and in intervention and comparison groups.

		**Intervention**				**Comparison**			
**Variable**	**Age**	**Pre**		**Post**		**Pre**		**Post**	
	**Group**	***n***	***M (SD)***	***n***	***M (SD)***	***n***	***M (SD)***	***n***	***M (SD)***
S1	8–11	1,386	4.87 (0.81)	1,386	4.94 (0.81)	915	4.83 (0.83)	915	4.87 (0.82)
	12–15	1,051	4.69 (0.76)	1,051	4.66 (0.76)	938	4.78 (0.74)	938	4.74 (0.77)
S2	8–11	1,379	4.10 (1.06)	1,379	4.03 (0.99)	911	4.03 (0.99)	911	4.09 (0.00)
	12–15	1,046	3.99 (0.87)	1,046	4.06 (0.87)	935	4.13 (0.90)	935	4.15 (0.88)
S3	8–11	1,371	4.13 (1.10)	1,371	4.11 (1.11)	898	4.04 (1.05)	898	4.06 (1.08)
	12–15	1,044	3.71 (0.99)	1,044	3.69 (1.04)	928	3.84 (1.02)	928	3.79 (1.06)
R1	8–11	1,371	4.84 (0.88)	1,371	4.86 (0.86)	907	4.76 (0.88)	907	4.80 (0.86)
	12–15	1,043	4.57 (0.82)	1,043	4.51 (0.83)	931	4.61 (0.85)	931	4.59 (0.87)
R2	8–11	1,373	4.80 (0.92)	1,373	4.76 (0.93)	905	4.69 (0.93)	905	4.69 (0.91)
	12–15	1,043	4.45 (0.86)	1,043	4.41 (0.85)	926	4.44 (0.85)	926	4.52 (0.91)
Problem	8–11	1,033	0.95 (3.68)	1,033	1.34 (3.32)	748	0.99 (3.34)	758	1.24 (3.82)
Behaviour^a^	12–15	877	0.61 (5.05)	877	1.29 (5.91)	758	0.19 (4.85)	758	0.63 (5.85)

*S1, Self-awareness; S2, Self-management; S3, Social awareness; R1, Relationship skills; R2, Responsible decision making*.

a *Calculated from z-scores*.

### SEL in Age Group 1 (8–11 Years Old)

Table 7 shows that the results of repeated measures GLM regarding *Self-awareness* (S1) in younger age group (8–11-year old) indicated no significant change across both groups [*F*_(1, 299)_ = 7.552, *p* = 0.12, partial η^2^ = 0.028]. In addition, no statistically significant interaction between the training (i.e., pre- and post-test) and the group [*F*_(1, 2299)_ = 0.523, *p* = 0.47, partial η^2^ = 0.000] could be found. However, when examining the intervention group and comparison group separately it was found that the change was significant in the intervention group [*F*_(1, 2299)_ = 7.58, *p* = 0.006, partial η^2^ = 0.003] but not in the comparison group [*F*_(1, 2299)_ = 1.702, *p* = 0.192, partial η^2^ = 0.001].

In the *Self-management* (S2) there was a significant positive change across intervention and comparison groups [*F*_(1, 2288)_ = 8.992, *p* = 0.006, partial η^2^ = 0.003]. However, the interaction between the training and the group was not significant [*F*_(1, 2288)_ = 0.136, *p* = 0.712, partial η^2^ = 0.000]. When investigating the intervention group and the comparison group separately, it was found that the change was significant in the intervention group [*F*_(1, 2288)_ = 7.13, *p* = 0.008, partial η^2^ = 0.003], but not in the comparison group [*F*_(1, 2299)_ = 2.871, *p* = 0.09, partial η^2^ = 0.001].

In the *Social awareness* (S3) there were no differences across the groups [*F*_(1, 2267)_ = 0.004, *p* = 0.951, partial η^2^ = 0.000] nor between the training and the group [*F*_(1, 2267)_ = 0.324, *p* = 0.569, partial η^2^ = 0.000] were found. No significant changes between the measuring points were found in the intervention group [*F*_(1, 2267)_ = 0.162, *p* = 0.687, partial η^2^ = 0.000] or in the comparison group [*F*_(1, 2267)_ = 0.165, *p* = 0.685, partial η^2^ = 0.000].

The results regarding *Relationship skills* (R1) showed no changes across both groups [*F*_(1, 2276)_ = 1.70, *p* = 0.193, partial η^2^ = 0.001] or between the training and the group [*F*_(1, 2276)_ = 0.330, *p* = 0.566, partial η^2^ = 0.000]. Changes between pre- and post-test in both intervention group [*F*_(1, 2276)_ = 0.333, *p* = 0.566, partial η^2^ = 0.000] and in the comparison group [*F*_(1, 2276)_ = 1.464, *p* = 0.226, partial η^2^ = 0.001] were not significant.

No significant changes [*F*_(1, 2276)_ = 0.805, *p* = 0.370, partial η^2^ = 0.000] were found across both groups in *Responsible decision making* (R2). In addition, no change was found between the training and the group [*F*_(1, 2276)_ = 0.946, *p* = 0.331, partial η^2^ = 0.000]. Further, the changes between the measuring points remained non-significant in both the intervention group [*F*_(1, 2276)_ = 2.200, *p* = 0.138, partial η^2^ = 0.001] and in the comparison group [*F*_(1, 2276)_ = 0.002, *p* = 0.124, partial η^2^ = 0.001].

### SEL in Age Group 2 (12–15 Years Old)

According to the results of the repeated measures GLM *Self-awareness* (S1) among older students (12–15 years old) no significant change was found across the groups [*F*_(1, 1987)_ = 3.737, *p* = 0.053, partial η^2^ = 0.002]. In addition, the change between the training and the group remained non-significant [*F*_(1, 1987)_ = 0.001, *p* = 0.971, partial η^2^ = 0.000] as well as the changes between the measuring points in both the intervention group [*F*_(1, 1987)_ = 2.055, *p* = 0.152, partial η^2^ = 0.001] and in the comparison group [*F*_(1, 1987)_ = 1.703, *p* = 0.192, partial η^2^ = 0.001] (see Table 7).

In the *Self-management* (S2) the difference across groups was significant [*F*_(1, 1979)_ = 5.364, *p* = 0.021, partial η^2^ = 0.003]. However, the change between the measuring point and the group [*F*_(1, 1979)_ = 1.605, *p* = 0.205, partial η^2^ = 0.001] was non-significant. Further investigations revealed positive significant change in the intervention group [*F*_(1, 1979)_ = 6.800, *p* = 0.009, partial η^2^ = 0.003] but not in the comparison group [*F*_(1, 1979)_ = 0.521, *p* = 0.470, partial η^2^ = 0.000].

No significant changes were found in the differences of *Social awareness* (S3) Across groups [*F*_(1, 1970)_ = 1.894, *p* = 0.169, partial η^2^ = 0.001] or between the training and the group [*F*_(1, 1970)_ = 0.673, *p* = 0.412, partial η^2^ = 0.000]. No significant changes between measuring points in the intervention group [*F*_(1, 1970)_ = 0.164, *p* = 0.685, partial η^2^ = 0.000] or in the comparison group [*F*_(1, 1979)_ = 2.278, *p* = 0.131, partial η^2^ = 0.001].

The results regarding *Relationship skills* (R1) showed a significant change across groups [*F*_(1, 1972)_ = 4.532, *p* = 0.033, partial η^2^ = 0.002]. However, the interaction between the training and the group was non-significant [*F*_(1, 1972)_ = 0.862, *p* = 0.353, partial η^2^ = 0.000]. The significant negative development of the intervention group was found between the measuring point [*F*_(1, 1972)_ = 4.954, *p* = 0.026, partial η^2^ = 0.003] whereas no development was found in the comparison group [*F*_(1, 1972)_ = 0.682, *p* = 0.409, partial η^2^ = 0.000].

The results of repeated measures GLM regarding *Responsible decision making* (R2) indicated a significant change across both groups [*F*_(1, 1967)_ = 1,972, *p* = 0.16, partial η^2^ = 0.001]. However, no statistically significant interaction between the training (i.e., pre- and post-test) and the group [*F*_(1, 1967)_ = 0.499, *p* = 0.48, partial η^2^ = 0.000] could be found. However, when examining the intervention group and comparison group separately it was found that there were no significant differences between measurements in the intervention group [*F*_(1, 1967)_ = 2.368, *p* = 0.124, partial η^2^ = 0.001] or in the comparison group [*F*_(1, 1967)_ = 0.230, *p* = 0.632, partial η^2^ = 0.000].

### Problem Behavior in Age Group 1 (8–11-Years Old)

In the group of younger students the results of repeated measures GLM revealed a significant change across the intervention and the comparison groups [*F*_(1, 1779)_ = 11.819, *p* = 0.001, partial η^2^ = 0.007] in Problematic behavior. However, the interaction between the training and the group was not found significant [*F*_(1, 1779)_ = 0.620, *p* = 0.431, partial η^2^ = 0.000]. However, when investigating the differences between measurements in intervention and comparison group separately there was a statistical positive development as a reduction of problem behavior in the intervention group [*F*_(1, 1779)_ = 10.628, *p* = 0.001, partial η^2^ = 0.006] but not in the comparison group [*F*_(1, 1779)_ = 3.028, *p* = 0.0082, partial η^2^ = 0.002].

### Problem Behavior in Age Group 2 (12–15-Years Old)

The results of the older students of the Problematic behavior showed that across groups there was a significant change over time [*F*_(1, 1663)_ = 19.151, *p* < 0.001, partial η^2^ = 0.012] whereas the interaction between the training and the group was non-significant [*F*_(1, 1663)_ = 0.937, *p* < 0.333, partial η^2^ = 0.001]. The significant negative development between measuring points was found both in the intervention group [*F*_(1, 1663)_ = 15,401, *p* < 0.000, partial η^2^ = 0.009] and in the comparison group [*F*_(1, 1663)_ = 5,404, *p* < 0.020, partial η^2^ = 0.003].

To conclude, the interactions of the time (pre and post) and group (intervention and comparison) were not significant showing that the effect of the intervention was vague. Pairwise comparisons showed some statistically significant both positive and negative changes in the intervention group, even when the change in the comparison group remained non-significant (i.e., younger students' Self-awareness, Self-management and Problem behavior and older students' Self-management and Relationship skills).

Due to the sensitive nature of the sum variable, all the participating countries were tested as one group. Univariate analyses of Variance was used to study the possible effect of the intervention. Table 8 shows that no statistically significant change in students' problem behavior was found during the intervention.

**Table 8:** Change in a sum variable called *problem behavior* that combined items concerning bullying, substance abuse and truancy.

	**Intervention**			**Comparison**			**Intcom**		
	***n***	**Mean**	**Std Dev**	***n***	**Mean**	**Std Dev**	***F*** **(df)**	***p***	***d***
Problem behavior	1,963	0.04	0.28	1,548	0.04	0.29	0.54 (1, 3,510)	0.46	0

*Intcom, between subjects effects*.

## Discussion

The present study investigated the effectiveness of the toolkit designed for assessing social and emotional skills in school. The main results showed that there was no statistically significant change in the answers between pre- and post-test given by the teachers. For the students no significant changes were found when looking at the results country by country. However, when looking at the students all together, there was a statistically significant positive change in the reports given in age group 1 in self-awareness and in self-management in the intervention group. No statistically significant change could be found in the comparison group. In the age group 2 there was a statistically significant negative development in terms of relationship skills in the intervention group whereas no statistically significant change could be found in the comparison group. In responsible decision making a significant change was found across both intervention and comparison group so it cannot be traced back to the intervention.

A similar development was found in problem behavior. In age group 1 a statistically significant positive development was found in the intervention group but not in the comparison group. In age group 2 no significant change was found.

Despite the fact that the teachers' reported competences and experienced importance of SEL did not change, there was still some change in the intervention group of the students that did not occur in the comparison group. The target of the intervention was to change assessment practices to assess competencies of the students rather than their factual knowledge. It is quite typical that the assessment is “the tail that wags the dog.” i.e., by changing assessment practices, we may be able to change the students' behavior and even their ways of thinking. However, there is some research suggesting that the change in assessment practices may not result in desired statistically significant outcomes concerning students' motivation and achievement (Yin et al., 2008). Formative assessment as a tool for learning may help the students to study for assessment or and change their goals accordingly (Dann, 2014). It was therefore possible that change took place among the students regardless of lack of change among their teachers.

When we combined the countries, it appeared that there was favorable development in the intervention in terms of the experienced self-awareness and self-management of the younger age group (8–11). In the older age group (12–15), only the experienced self-awareness increased over time in the intervention group, but not in the comparison group. In this age group, the relationship skills even decreased in the intervention group. It appeared that the intervention had some added value especially among the younger participants that could not be explained based on the development during the 6 months. However, considering that the interactions were not significant, we cannot conclude that the intervention was the reason for the changes among the students.

Because the intervention was about assessing the SEL skills, it is possible that it only raised the self-awareness of the students but did not quite reach the level of improving their skills. The puberty may have had an effect on the teenager group, it may have made it difficult for them to manage themselves and becoming aware of their self-management problems may have made the experience their relations skills even lower than before the intervention. Looking at these results makes sense: starting to assess and reflect one's SEL skills is the first step toward starting to develop such skills. It is possible that teachers and their students learned from the intervention in the way that the students started to be aware of their own behavior. This is a good start for future learning of these skills, and it is valuable to further continue the efforts in teaching SEL more concretely.

The research methods should also be reflected on. The research sample was not randomized and it appears the analysis method including the questionnaire just did not capture participants' learning in this case. Despite the satisfactory psychometric properties of the questionnaire, the instrument may have been too long and in some respect inappropriate for the targeted age groups of the students. It may also be that the questionnaire despite the back-translation process did not yield to different cultures due to translation difficulties regarding cultural expressions. However, on behalf of the teachers, probably the problem did not lie in the measurement instruments, because the same instruments had previously captured teachers' development of SEL in different countries. These previous studies were on well-established and well-structured instructional procedures such as Lions Quest (Talvio et al., 2016, 2019). Such programs do not only aim at assessing SEL, but also provide concrete tools for developing the related skills. Because there could have been even 6 months between the pre- and post-tests, there may have been other development taking place in the students that could not be differentiated from the effects of the intervention.

Collecting post-data right before the end of the school year might have affected the answers of both the teachers and their students. For teachers the measurement point may have been too wide apart so that the contents possibly learned during the intervention had been forgotten due to the heterogeneous nature of the teachers' workload. Teachers might also be busy with evaluation processes as well as different school activities concerning the end of the school year. At the same time, students might be disengaged and focused on the upcoming summer holiday. Therefore, it is possible that teachers and their students learned more than what the post-test showed.

Of course, it is also possible that the interventions were not effective at short term. They were newly developed and the time for their testing and their further development might have been too short. Indeed, many established trainings (for example Lions Quest) for promoting positive growth and well-being (Lions Qust, Youth Effectiveness Training) have been available over 30 years, during which time they have been continuously developed, based on the feedback of teachers and their students. Accordingly, developing SEL interventions might need more time and continuous interaction between the program developers, practitioners and policy makers. In addition, in the studies of expertise, it takes time to proceduralize the knowledge into skills (e.g., Ericsson and Ward, 2016). Some studies (Baartman and De Bruijn, 2011) suggest that transformative integration of knowledge, skills and attitude requires critical self-reflection and openness to change. From this point of view it is possible the measuring points were too close to each other for the teachers to become experts in teaching SEL and accordingly the students to gain knowledge (from the teachers) that would have then transformed into skills with sufficient amount of practice.

It was important, though, that the research partner was independent of those who carried out the interventions. This applies especially in the case like this, where the results are not quite desirable. We think, however, that this is also an important research result: there were no obvious changes in the actual relationship skills by using this kind of intervention design. More work is needed to develop the interventions further, from assessing the SEL skills into systematically training them during a longer period of time. Acquiring social and emotional skills and learning to apply them in classroom situations and in teaching is a time consuming process which is not likely to happen over a short period of time. It would be also important to test the actual skills in different contexts and with more fine-grained research instruments.

More contextual information about specific schools would have been enriching, but the current ethical and GDPR regulations of EU did not allow us to risk the anonymity of the participants. Some schools were so small that there were only two teachers. Revealing the school name would have also revealed their identity. Large-scale studies have their benefits, but may hide some important contextual variation. However, participating countries are preparing additional analysis regarding the data content concerning exclusively the teachers and students of their own country. In addition, the qualitative research based on the monitoring procedures of the intervention is being conducted at the University of Latvia (in progress) and it may reveal more about the contextual aspects of the interventions.

We shall also see, whether some starting points of the teachers and students would have resulted in so called ATI (aptitude-treatment interactions). Even though all the participants were provided with equal opportunities to gain as much as their abilities allow, the individual starting levels of the participants may affect the results (Merrill, 1975). Such questions were not included in the research problems of the project goals, but we shall be able to use the data in order to test some new hypotheses. In all, the project produced important new information of the complexity that has arisen in many investigations of SEL issues (Collie et al., 2017; Lawson et al., 2019) and inspired many new research questions.

## Conclusions

Even the most popular SEL approaches used at school do not always present strong evidence of effectiveness in learning SEL (Corcoran et al., 2018). Even though the use of quasi-experimental design with pre-validated questionnaires has been practical in assessing many well-known established SEL interventions, the effect of the intervention may still be difficult to capture (Ura et al., 2020). We found out that already starting to focus on the assessment of SEL appeared to change the participants' self-awareness regardless of the age group. The younger participants even learned some self-management skills that were more difficult for the teenagers. This may indicate that interventions should be started before the stormy phase of puberty. However, regarding new SEL interventions more contextual and qualitative approach in investigations would probably give more understanding of how the interventions could be further developed.

## Data Availability Statement

The data analyzed in this study is subject to the following licenses/restrictions: GDPR regulations were taken into account. All data that might enable the identification of an individual participant was deleted and replaced by a participant number. The data file matching the participant codes for matching the pre- and post-test answers is saved in a separate file at the University of Helsinki. Requests to access these datasets should be directed to lauri.hietajarvi@helsinki.fi.

## Ethics Statement

The studies involving human participants were reviewed and approved by Ethical Review Board in the humanities and social and behavioral sciences of the University of Helsinki. Written informed consent to participate in this study was provided by the participants' legal guardian/next of kin.

## Author Contributions

MB: leading writer. MT: post-doc researcher of the project and contributor to data analysis as well as supervisor of MB and contributor to the writing process. LH: data analyses. IB, VC, EC, IR, BM, and ST: collecting of data and contributing to writing. FC and MK: contributing to writing. MF: contributing to composing the intervention and writing. VO: arranging the intervention in Italy and contributing to writing. DŠ: contributing to the content of the intervention and to the writing. KL: supervising the project and MB as well as contributing to writing. All authors contributed to the article and approved the submitted version.

## Conflict of Interest

The authors declare that the research was conducted in the absence of any commercial or financial relationships that could be construed as a potential conflict of interest.

## Publisher's Note

All claims expressed in this article are solely those of the authors and do not necessarily represent those of their affiliated organizations, or those of the publisher, the editors and the reviewers. Any product that may be evaluated in this article, or claim that may be made by its manufacturer, is not guaranteed or endorsed by the publisher.
